# Green Synthesis of Chitosan-Capped Gold Nanoparticles Using *Salvia officinalis* Extract: Biochemical Characterization and Antimicrobial and Cytotoxic Activities

**DOI:** 10.3390/molecules28237762

**Published:** 2023-11-24

**Authors:** Faisal Al-Sarraj, Ibrahim Alotibi, Majid Al-Zahrani, Raed Albiheyri, Mashail A. Alghamdi, Nada M. Nass, Sawsan Abd-Ellatif, Raafat T. M. Makhlof, Mohammad A. Alsaad, Bayan H. Sajer, Hazem S. Elshafie

**Affiliations:** 1 Department of Biological Sciences, Faculty of Science, King Abdulaziz University, Jeddah 21589, Saudi Arabia; falsaraj@kau.edu.sa (F.A.-S.); ralbiheyri@kau.edu.sa (R.A.); mamalgamdi2@kau.edu.sa (M.A.A.); nmnass@kau.edu.sa (N.M.N.); bsajer@kau.edu.sa (B.H.S.); 2 Health Information Technology Department, Applied College, King Abdulaziz University, Jeddah 21589, Saudi Arabia; ialotibi@kau.edu.sa; 3 Biological Science Department, College of Science and Art, King Abdulaziz University, Rabigh 21911, Saudi Arabia; maalzahrani4@kau.edu.sa; 4 Centre of Excellence in Bionanoscience Research, King Abdulaziz University, Jeddah 21589, Saudi Arabia; 5 Bioprocess Development Department, Genetic Engineering and Biotechnology Research Institute (GEBRI), City of Scientific Research (SRTA-City) and Technological Applications, Alexandria 21934, Egypt; sabdellatif@srtacity.sci.eg; 6 Department of Parasitology, Faculty of Medicine, Umm Al Qura University, Makkah 21955, Saudi Arabia; rtmakhlof@uqu.edu.sa (R.T.M.M.); masaad@uqu.edu.sa (M.A.A.); 7 Department of Parasitology, Faculty of Medicine, Minia University, Minia 61511, Egypt; 8 School of Agricultural, Forestry, Food and Environmental Sciences (SAFE), University of Basilicata, Via dell’Ateneo Lucano 10, 85100 Potenza, Italy

**Keywords:** multidrug-resistant bacteria, chitosan–capping AuNPs, cell viability, antibacterial, antioxidants, anticancer, *S. officinal* extract, composite nano characterization

## Abstract

Increasing antimicrobial resistance to the action of existing antibiotics has prompted researchers to identify new natural molecules with antimicrobial potential. In this study, a green system was developed for biosynthesizing gold nanoparticles (BAuNPs) using sage (*Salvia officinalis* L.) leaf extract bioconjugated with non-toxic, eco-friendly, and biodegradable chitosan, forming chitosan/gold bioconjugates (Chi/BAuNPs). Characterization of the BAuNPs and Chi/BAuNPs conjugates takes place using transmission electron microscopy (TEM), X-ray spectra, Fourier transform infrared (FT-IR) spectroscopy, and zeta potential (Z-potential). The chemical composition of *S. officinalis* extract was evaluated via gas chromatography/mass spectrometry (GC/MS). This study evaluated the antioxidant and antimicrobial activities of human pathogenic multidrug-resistant (MDR) and multisensitive (MS) bacterial isolates using the agar diffusion method. Chi/BAuNPs showed inhibition of the MDR strains more effectively than BAuNPs alone as compared with a positive standard antibiotic. The cytotoxicity assay revealed that the human breast adenocarcinoma cancer cells (MCF7) were more sensitive toward the toxicity of 5-Fu + BAuNPs and 5-Fu + Chi/BAuNPs composites compared to non-malignant human fibroblast cells (HFs). The study shows that BAuNPs and Chi/BAuNPs, combined with 5-FU NPs, can effectively treat cancer at concentrations where the free chemical drug (5-Fu) is ineffective, with a noted reduction in the required dosage for noticeable antitumor action.

## 1. Introduction

AMR, or antimicrobial resistance, represents a serious threat to our society. It occurs when multidrug-resistant bacteria (MDR) gain resistance to the available antimicrobials and/or chemotherapeutic medicines [1]. Specific resistance to the majority of antibiotics has emerged to date. Innovative and potent antibacterial strategies must be developed immediately [2]. There are two key issues that need to be resolved: first, there are no new classes of antibiotics; second, the time between the commercial release of new versions of existing antibiotics and the emergence of resistant bacterial strains is becoming shorter [3]. Due to rising drug resistance, antibiotics have recently been administered in higher doses, and the toxicity that has resulted has brought attention to the necessity of developing and defining novel and effective antimicrobial treatments [4,5,6,7]. Recently, there have been two models of in vitro and in vivo research; the creation of novel potential alternative antimicrobial agents to combat bacterial infections and for drug administration has been made possible by the development of nanotechnology [8,9]. It is possible to use nanoparticles safely in the medical field using the environmentally friendly method of biosynthesis [10,11]. Nanomaterials with antibacterial properties against human pathogenic bacteria and fungi include gold, silver, copper, selenium, titanium, zinc oxide, and magnesium oxide [12,13,14,15,16].

Due to their distinctive qualities, including customizable size, shape, surface properties, optical properties, biocompatibility, low cytotoxicity, and excellent stability, gold nanoparticles are among the most-often-utilized metals for biomedical applications [17,18,19].

Many potential medicinal uses of AuNPs, including as drugs and gene delivery, have been investigated [20,21]. The synthesis, stabilization, and functionalization of AuNPs are major areas of scientific focus [22]. After repeated treatments, the body may accumulate AuNPs at toxic levels [23]. As a result, the majority of AuNPs research is still in the preclinical stage [24]. It is recommended to utilize non-toxic reagents to increase the biocompatibility of AuNPs [25]. These ideas have given rise to a number of papers that proposed novel methods for synthesizing green AuNPs using green reduction and protection agents. These agents’ function is to adsorb onto the surface of the freshly created NPs in order to stop further growth and particle aggregation [26,27]. Plants have provided the majority of these reducing and stabilizing substances [14,28]. Several protective agents are frequently employed to stabilize and cap the nanoparticles. Organic, inorganic, or complex systems found in nature can be used as capping agents. Chitosan is a biopolymer composed of polysaccharides that exhibits remarkable biocompatibility, biodegradability, and low toxicity [29,30,31,32].

A wide range of complex nutrients and bioactive metabolites, including alkaloids, phenolic compounds, proteins, fatty acids, carbs, and amino acids, are found in plant extracts and are essential for the production, reduction, and capping of nanoparticles [33,34]. The largest genus of plants in the *Lamiaceae* family, *Salvia officinalis* L., contains about 900 species [35]. Due to this plant’s antibacterial, anticancer, antifungal, and anti-inflammatory characteristics, it has, for a long time, been used in traditional medicine to treat illnesses such as colds, gastrointestinal problems, bronchitis, malignancies, and tuberculosis [36]. 

In previous studies, the *Salvia officinalis* herbal plant has been chosen for the synthesis of gold nanoparticles, and the indoor work shows that this plant has been widely used in pharmacognosy [37,38,39,40]. AgNPs were successfully biosynthesized using *Salvia officinalis* leaf extract as an efficient biological coating and a stabilizing agent, nanomaterials showing significant antibacterial activities against *Salmonella typhimurium*, *Pseudomonas aeruginosa*, *Staphylococcus aureus*, and *Escherichia coli* [41]. The green synthesis of silver nanoparticles (SVAgNPs), obtained using aqueous extracts of *Salvia verticillata* with biological properties, showed promising antimicrobial potential, antioxidant activity, and significant cytotoxic activity (IC_50_ 31.50 µg/mL) against human colon cancer HCT-116 cell lines; moreover, SVAgNP showed pronounced antibacterial activity (MIC < 39.1 µg/mL) for most of the tested bacterial species, i.e., *Micrococcus lysodeikticus* ATCC 4698, *Enterococcus faecalis* ATCC 29212, *Escherichia coli* ATCC 25922, *Klebsiella pneumonia* ATCC 70063, *Pseudomonas aeruginosa* ATCC 10145, *Bacillus cereus* ATCC 10876, *Bacillus subtilis* ATCC 6633, *Salmonella enteritidis* ATCC 13076, *Salmonella typhimurium* ATCC 14028, *Staphylococcus epidermidis* ATCC 12228, and *Staphylococcus aureus* ATCC 25923 [42]. Furthermore, the antifungal activity of biosynthesized ZnONPs using aqueous leaf extract of *S. officinalis* was determined against different clinical *Candida albicans* isolates that showed significant growth inhibition to the tested clinical *C. albicans* isolates [43]. 

The main objective of the present work was to biosynthesize environmentally friendly gold nanoparticles in an aqueous extract of *S. officinalis* (BAuNPs), to prepare non-toxic and biodegradable chitosan-coated, green-synthesized gold nanoparticle conjugates (Chi/BAuNPs), and to characterize their different properties, such as their synthesis rate, yield, stability, crystallite size, and morphology. Next, we evaluated the antioxidant and antibacterial activities of BAuNPs and Chi/BAuNPs composites against multidrug-resistant bacteria such as *Pseudomonas aeruginosa* (*P. aeruginosa*), *Escherichia coli*, (*E. coli*), *Klebsiella pneumonia* (*K. pneumonia*), and *Staphylococcus aureus* (*S. aureus*). In addition, we evaluated, in vitro, the cytotoxic effect of the free 5-Fu, or in combination with diverse concentrations of BAuNPs and Chi/BAuNPs, against human breast adenocarcinoma cells (MCF7) and non-malignant human fibroblast cells (HFs).

## 2. Results

### 2.1. Chemical Analysis of Aqueous Extract from S. officinalis Leaves 

#### 2.1.1. Chemical Composition

**Figure 1:**
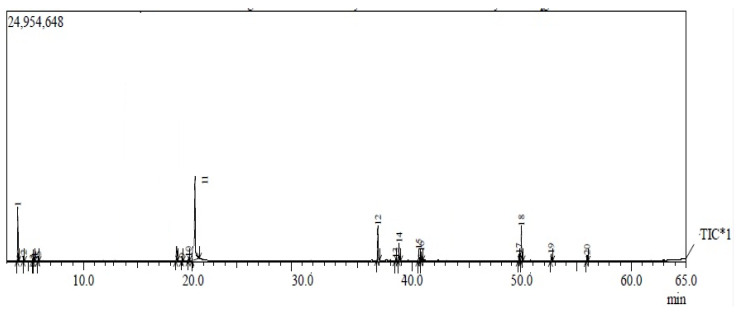
GC-MS of aqueous extract from leaves of *Salvia officinalis*.

The percentages and retention times (RI) of the chemical compounds in the aqueous extract of *S. officinalis*, identified using GC-MS analysis, are shown in Figure 1 and summarized in Table 1. The main fractions detected were richness in salvianolic acid (20.69%), rosmarinic acid (11.42%), caffeic acid hexoside (10.19%), luteolin-7-o-rutinose (9.78%), apigenin (8.66%), apigenin-7-glucoside (8.12%), dicaffeoylquinic acid (7.76%), carvacrol (6.03%), methyl rosmarinate (4.14%), thujone (2.37%), salvigenin (2.0%), ferulic acid derivative (1.88%), and camphor (0.53%) (Table 1), which have many bioactive properties, including antimicrobial, antitumor, and antioxidant activities. Many medicinal plants have a natural terpene.

#### 2.1.2. Phenolic Content

The phenolic content in an aqueous extract from the leaves of *S. officinalis* was 173.3 ± 27.61 mg GAE/g of extract. Since phenolic acids were found to be the major class of phenolic compounds for all the studied species, salvianolic acid, rosmarinic, ferulic, p-coumaric chlorogenic, and caffeic acids were frequent occurrences in *S. officinalis* plants [44,45,46], and, in vitro, they showed antioxidant activity [47]. The highest concentration of rosmarinic acid in *S. officinalis* was also reported by Farhat et al. [48].

### 2.2. Preparation of Green-Synthesized AuNPs Conjugates

Gold nanoparticle (BAuNPs) conjugates were prepared through green-synthesized *S. officinalis* aqueous extract. The prepared BAuNPs were characterized using TEM, EDX, and FTIR, particle size was measured using zeta potential analysis, and they were further tested for their antioxidant, antibacterial, and anticancer properties.

### 2.3. Characterization of BAuNPs 

#### 2.3.1. UV-Vis Spectroscopy

**Figure 2:**
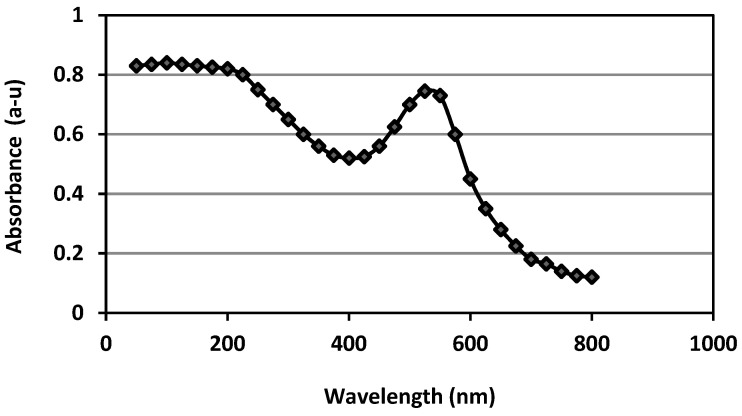
UV-Vis spectroscopy graphs of green-synthesized gold nanoparticles using aqueous extract from leaves of *S. officinalis* as a reducing agent.

The surface plasmon resonance (SPR) absorption spectra ranged from 100 to 800 nm, indicating that AuNPs were generated during overnight incubation. The SPR of the formed BAuNPs was at 530 nm, while the SPR absorption spectral ranged from 400 to 600 nm (Figure 2).

#### 2.3.2. The Transmission Electron Microscopy Analysis of BAuNPs

**Figure 3:**
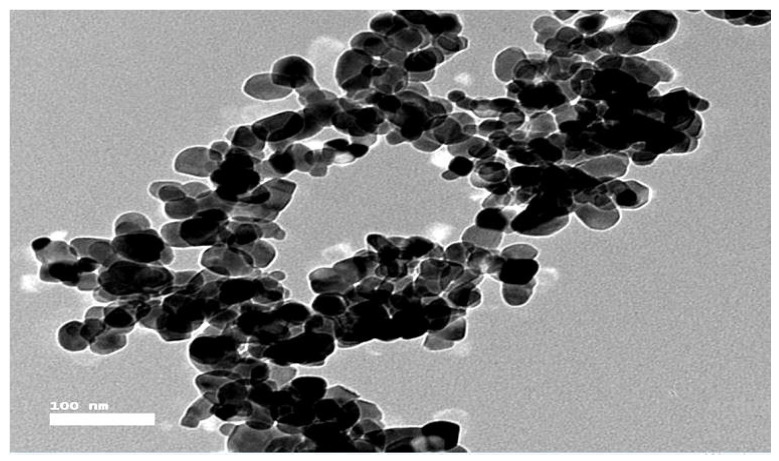
TEM graphs of BAuNPs in aqueous extract from leaves of *S. officinalis*.

The most effective technique for measuring the morphological structure and precise particle size of the green-synthesized BAugNPs produced from *S. officinalis* plant extracts is TEM (Figure 3). The TEM image shows spherical aggregated shapes with sizes in the range of 15 to 70 nm on average.

#### 2.3.3. EDX Analysis of BAuNPs

**Figure 4:**
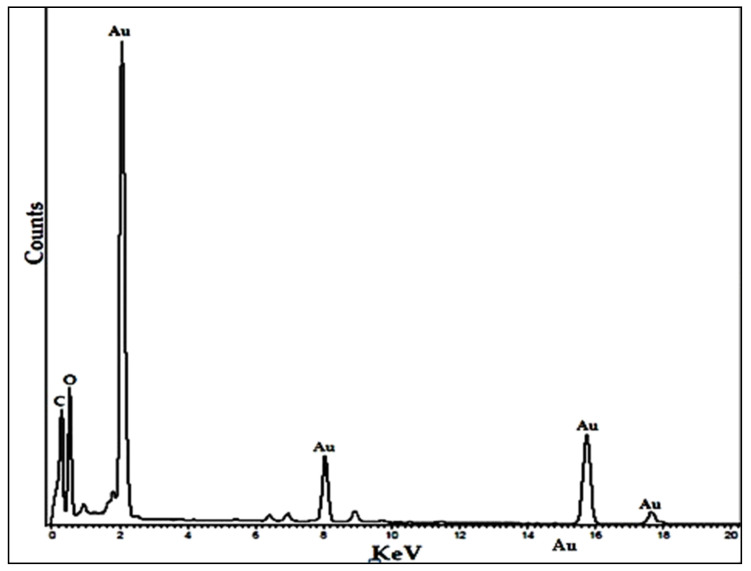
Energy-dispersive X-ray spectroscopy spectra of BAuNPs from the extracts of *S. officinalis*.

Energy-dispersive X-ray spectroscopy (EDX) analysis of green-synthesized gold nanoparticles confirmed the existence of gold ions in the TEM-investigated BAuNP. The EDX spectra were performed at 9.7, 11.3, and 23 Ke V (Figure 4), which are consistent with a previous study [24], and the EDX spectra revealed the existence of several well-defined peaks associated with the gold nanostructures (Au); the carbon (C) component peak is attributed to the TEM grid, and the detector window [49] and oxygen (O) peak may be due to traces of phytochemicals in the *S. officinalis* extract [50,51].

#### 2.3.4. The FT-IR Spectrum Analysis of BAuNPs

**Figure 5:**
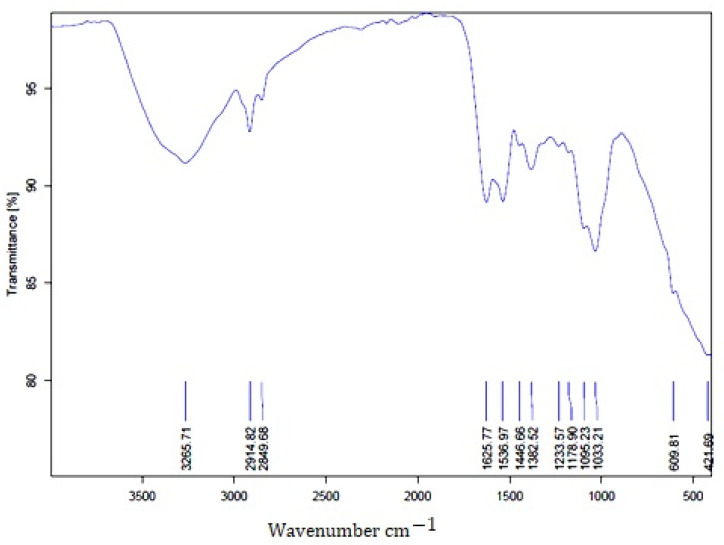
FT−IR analysis of BAuNPs.

Fourier transform infrared spectroscopy (FT-IR) spectrum analysis of green-synthesized *S. officinalis* BAuNPs was performed by reducing the molecular interaction and using the *S. officinalis* extract as a capping agent, which enhanced the formation and stabilized the BAuNPs (Figure 5). The FT-IR spectra of BAuNPs show many peaks of functional groups at different wave numbers (3265, 2914, 2849, 1625, 1536, 1446, 1382, 1233, 1178, 1095, 1033, 609, and 421 cm^−1^). The spectrum of the synthesized BAuNPs exhibited absorption bands at 2914 and 3265 cm^−1^. The stretched broad band observed at 3265 cm^−1^ absorbance and the band at 2914 cm^−1^ may correspond to the C-H stretching vibrations of the alkanes group and the aldehydic variable group, whereas the peak at 2849 cm^−1^ matched the vibration of the amino acid group (N-H). The band at 1625 cm^−1^ indicates the existence of a strong carbonyl (C=O) group of amide I, and the narrow peak at 1536 cm^−1^ reveals the presence of a strong carbonyl (C=O) group of amide II and the C=N stretching vibration group. However, the peak at 1446 cm^−1^ may be because of the –N-H group. The peak at 1233 cm^−1^ may be because of the –C-O group. The narrow peak at 1095 cm^−1^ can be assigned to the presence of C-N stretching vibrations of aliphatic amines. General bands and assignments of the FTIR spectra of *Salvia officinalis*, 609 cm^−1^ band (600–680 cm^−1^, -alkyne C-H bend), 1178^−1^ (1130–1190^−1^, -secondary amine –CH stretch), 1233^−1^ (1220–1270^−1^, -PO2-antisymmetric str), 1033 cm^−1^ and 1095 cm^−1^ (900–1200 cm^−1^, -C-O, C-C str., C-O-H, C-O-C def (carbohydrate), 1382 cm^−1^ (1370–1420 cm^−1^, -organic sulfate), 1446 cm^−1^ (1430–1470 cm^−1^, -methyl C-H asym/sym bend), 1625 cm^−1^ (1590–1680 cm^−1^, -C = C str), and 2849 (1845–1855,-C-H str. (sym) of CH2 from lipid acyl chains) are as described Pin references [52,53,54,55]. The 3265 band (3400–3200 cm^−1^, hydroxyl group (O–H) and H-bonded stretching) is characteristic of polyphenolic compounds [56].

#### 2.3.5. Particle Size Analysis

**Figure 6:**
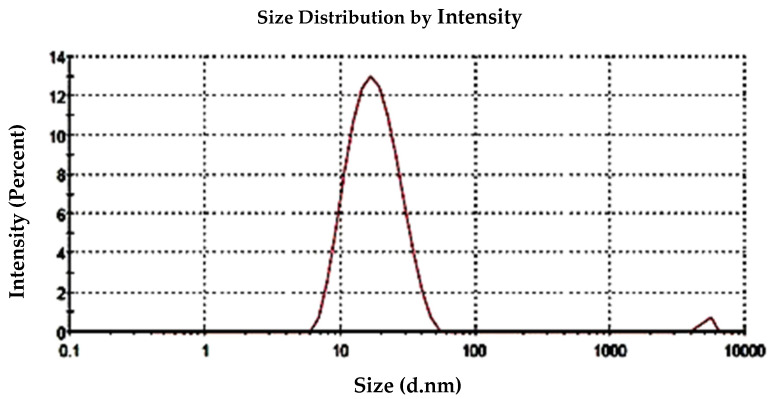
Histograms of the particle size distribution via zeta potential analysis of the green-synthesized BAuNPs composites using aqueous leaf extract of *S. officinalis* as a reducing agent.

Generally, it seems the role of adding the aqueous *S. officinalis* extract to the BAuNPs synthesized process is that of a reducing and capping agent that has a high content of bioactive compounds of polyphenols, flavonoids, and terpenoids fractions, accelerating the nucleation process and the formation of green-synthesized AuNPs at a small size. Therefore, adding the *S. officinalis* extract will lead to stopping the reducing reaction and result in a reduction in the particle size of the produced BAuNPs. The sizes of the obtained BAuNPs nanoparticles, measured using zeta potential as a mean value of the formed BAuNPs composite size, was 21.6 nm (Figure 6). The polydispersity index (PI) of the synthesized BAuNPs was 0.219.

### 2.4. DPPH and ABTS Scavenging Activities

**Figure 7:**
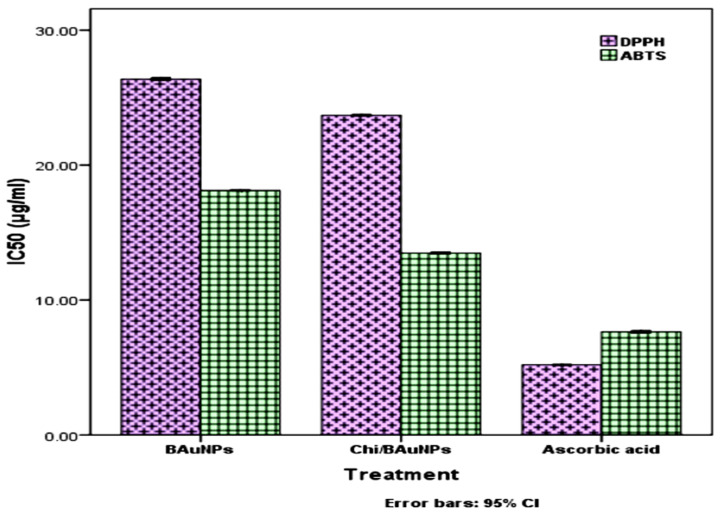
Half-maximal inhibitory concentration (IC_50_ values, μg/mL) was assessed using ascorbic acid as a positive control.

The radical scavenging activities of green-synthesized *S. officinalis* BAuNPs and chitosan-coated BAuNPs (Chi/BAuNPs) conjugates at different dilutions (100, 200, and 300 µg/mL) of nanoparticles were screened in vitro using DPPH (1,1-Diphenyl-2-picrylhydrazyl) radical scavenging activity and ABTS (*2*,2-azinobis-(3-ethylbenzothiazoline-6-sulfonicacid) radical cation photometric assays. DPPH scavenging activities are based on their reduction in the presence of an antioxidant as a donor compound. In this work, the inhibitory results of different concentrations of BAuNP and Chi/BAuNP on DPPH and ABTS free radicals are presented in Table 2, and IC_50_ values are recorded in Figure 7.

Overall, the scavenging abilities of Chi/BAuNPs and BAuNPs against DPPH and ABTS free radicals were dose-dependent since an increase in inhibition activities against both radicals was observed when increasing the concentrations of BAuNPs and Chi/BAuNPs conjugates. The maximum inhibition percentage of the ABTS radical was 53.15% and recorded at an application of 300 μg/mL Chi/BAuNPs, followed by 44.22% observed at 300 μg/mL of BAuNPs composites, as compared with the 67.26% recorded in 300 μg/mL ascorbic acid (positive control), while the minimum inhibition (17.65%) was observed at 100 μg/mL of green-synthesized BAuNPs composites (Table 2). The IC_50_ values of ABTS inhibition were 18.12 ± 0.02 μg/mL and 13.47 ± 0.026 μg/mL with the application of BAuNPs and Chi/BAuNPs composites, respectively, as compared with 7.64 ± 0.032 μg/mL in ASA (positive control) (Figure 7), while the maximum inhibition percentage of the DPPH radical was 69.84%, recorded at an application of 300 μg/mL of Chi/BAuNPs, followed by 51.08% observed at 300 μg/mL of BAuNPs composites, as compared with 81.05% recorded in 300 μg/mL ascorbic acid (positive control),while the minimum inhibition percentage was 22.60% at 100 μg/mL of green-synthesized BAuNPs composites (Table 2). The IC_50_ values of ABTS inhibition were 26.37 ± 0.035 μg/mL and 23.68 ± 0.68 μg/mL with the application of BAuNPs and Chi/BAuNPs composites, respectively, as compared with 5.2 ± 0.01μg/mL in ASA (positive control) (Figure 7).

### 2.5. Antibacterial Assay

**Figure 8:**
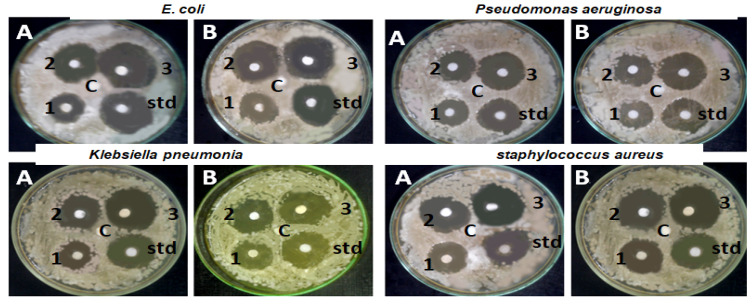
The antimicrobial effects of different concentrations—100 μg/mL (1), 200 μg/mL (2), and 300 μg/mL (3)—of *S. officinalis* BAuNPs (**A**) and Chi/BAuNPs (**B**) composites against four human pathogenic bacterial strains: *E. coli*, *P. aeruginosa*, *K. pneumonia*, and *S. aureus* in vitro, as compared with 10 μg/mL standard penicillin/streptomycin solution.

The antibacterial activity of various concentrations of *S. officinalis* green-synthesized BAuNPs and Chi/BAuNPs composites (100, 200, and 300 μg/mL) was evaluated against four human pathogenic and multidrug-resistant bacterial strains of *E. coli*, *P. aeruginosa*, *K. pneumonia*, and *S. aureus* in vitro, as compared with a 10 μg/mL penicillin/streptomycin standard antibiotic solution and DMSO as the negative control group of the study. Table 3 shows the diameter of the inhibition zone of BAuNPs and Chi/BauNPs composites. The results reveal antibacterial activity against four investigated bacterial strains. The antibacterial effect of nanoparticles was dose-dependent since an increase in antibacterial potential was associated with an increase in the concentration of BauNPs and Chi/BauNPs composites as compared to the positive control. In addition, *P. aeruginosa* and *S. aureus* bacteria were more susceptible to the green-synthesized BauNPs and Chi/BauNPs compared to *Klebsiella pneumonia* and *E. coli* strains (Figure 8); this may be due to the difference in bacterial cell wall structures [57]. The highest inhibition activities of Chi/AuNPs and BauNPs conjugate were 31, 30, 29, and 28 (mm) at 300 μg/mL of Chi/BauNPs against *S. aureus*, *P. aeruginosa*, *K. pneumonia*, and *E. coli* strains, respectively, followed by positive control inhibition 11 (mm) recorded at 100 μg/mL of BauNPs against the *E. coli* strain, as compared with positive control inhibition activity 33, 32, 31, and 30 (mm) against multidrug-resistant strains *P. aeruginosa*, *S. aureus*, *K. pneumonia*, and *E. coli*, respectively.

The main properties of the bioactive polymer chitosan are its non-toxicity, biodegradability, biocompatibility, low immunogenicity, and hemostatic properties [58,59,60]. By conjugating non-toxic, low-immunogenic, biodegradable, and biocompatible chitosan with bioactive AuNPs, their efficacy and stability will increase significantly, with a significant decrease in AuNP toxicity. Potara et al. [61] report that chitosan stabilizes AgNPs and inhibits agglomeration. Additionally, AgNPs receive a positive charge from chitosan, which improves their ability to attach to the negative charges found on bacterial cell surfaces. Saha et al. [62] state that chitosan improves the stability and performance of AuNP_S_ that is biosynthesized. In contrast to biosynthesized AgNP_S_, biosynthesized Chi-AgNPs have greater antibacterial action against pathogenic bacteria according to Shinde et al.’s [63] investigation into the antibacterial activity of biosynthesized AgNPs and Chi-AgNPs. Additionally, they discovered that normal cells do not exhibit any toxicity from Chi-AgNPs. The antibacterial activity of Ch-AuNPs was examined by Fuster et al. [52] against two Gram-positive bacterial strains—methicillin-resistant *S. aureus* ATCC 43300 and methicillin-sensitive *S. aureus* ATCC 29213—and Gram-negative *E. coli* ATCC 25922, a clinical isolate of *E. coli* 11046 (CI-EC). Ch-AuNPs demonstrated noteworthy antibacterial efficacy against every pathogenic strain examined, indicating that they may be a viable option for mitigating bacterial infections in the future [64].

The inhibitory activities of green-synthesized BAuNPs and Chi/BAuNPs composites in DMSO were evaluated according to MIC and MBC values against various human pathogenic strains as compared with the antibacterial activities of standard antibiotics (Table 4). Three Gram-negative bacteria (*P. aeruginosa*, *E. coli*, and *K. pneumonia*) and three Gram-positive bacteria (*S. aureus*) were investigated via turbidity. According to the results presented in Table 4, the Chi/BAuNPs composites exhibited higher antimicrobial activities than BAuNPs conjugate against *P. aeruginosa*, *E. coli*, *K. pneumonia*, and *S. aureus*, respectively, with minimal inhibitory concentration (MIC) values of 563, 394, 453, and 711 g/mL, while the MIC values of BAuNPs against these bacteria were 984, 648, 843, and 1352 g/mL, respectively. Furthermore, the minimum bactericidal concentration (MBC) values presented in Table 4 indicated that the bactericidal activities of Chi/BAuNPs composites were much higher than those of BAuNPs against *P. aeruginosa*, *E. coli*, *K. pneumonia*, and *S. aureus.*

### 2.6. In Vitro Anticancer Activity of BAuNPs and Chi/BAuNPs Composites

**Figure 9:**
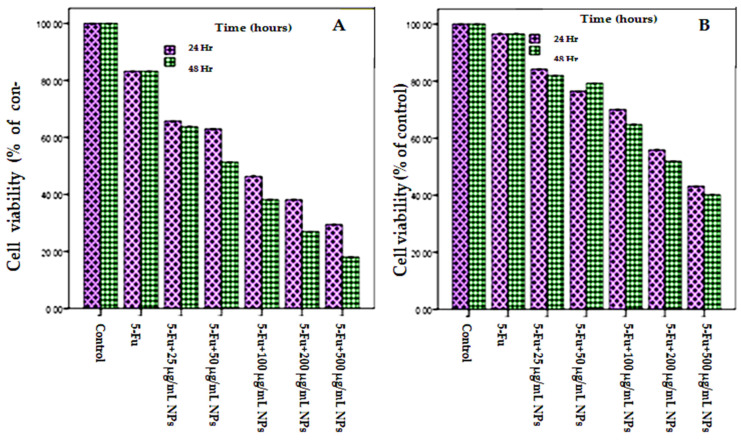
Dose-dependent cytotoxicity on the proliferation of human breast adenocarcinoma (MCF7) Cell line (**A**), and non-malignant human fibroblast (HFs) cell line (**B**) at 5-Fu (8 µg/mL) or 5-Fu combined with various concentrations (0, 25, 50, 100, 200, and 500 µg/mL) of NPs for 24, and 48 h exposure time. Values represent the means of three replicates ± SE. IC_50_: the half-maximal inhibitory concentration. (N.B.) significant at *p* ≤ 0.05, compared with negative control and 8 µg/mL free 5-Fu (positive control).

The cytotoxicity effect of 5-Fu + BAuNPs and 5-Fu + Chi/BAuNPs composites was determined in vitro against human breast adenocarcinoma cells (MCF7) (Figure 9A) and non-malignant human fibroblast cells (HFs) (Figure 9B) exposed to various concentrations (0, 25, 50, 100, 200, and 500 μg/mL) using the MTT assay in comparison with an 8 μg/mL 5-Fu positive control. The cell viability rate of MCF7 cells is represented in Figure 9A, showing a decrease with increasing concentrations of 5-Fu + BAuNPs and 5-Fu + Chi/BAuNPs composites. The results obtained from the cytotoxicity assay revealed that the MCF7 cancer cells were more sensitive toward the toxicity of 5-Fu + BAuNPs and 5-Fu + Chi/BAuNPs composites compared to the non-malignant HFs cells; both BAuNPs and Chi/BAuNPs composites exhibited a concentration-dependent cytotoxic (cell mortality) effect on MCF7 cells, and the maximum cytotoxic effect was observed at 17.99 ± 0.27% cell viability observed at 5-Fu + 500 μg/mL Chi/BAuNPs, followed with 26.93 ± 0.015% and 29.42 ± 0.025% cell viability rates at 5-Fu + 200 μg/mL Chi/BAuNPs and 5-Fu + 500 μg/mL BAuNPs, respectively, while the lowest toxicity effect against MCF7 83.23 ± 0.03% cell viability was observed at free 8 5-Fu μg/mL as compared with the 100% cell viability rate in negative control (DMSO). The amounts of 5-Fu + Chi/BAuNPs and 5-Fu + BAuNPs composites required to decrease the cell viability rate of MCF7 cancer cells to 50% of the initial population (IC_50_) were 34.1932 μg/mL and 46.32 μg/mL, respectively (Figure 9A).

The results obtained from the cytotoxicity assay of 5-Fu + BAuNPs and 5-Fu + Chi/BAuNPs composites against non-malignant HFs cells (Figure 9B) showed that both BAuNPs and Chi/BAuNPs composites had no significant toxicity at concentrations of 8 μg/mL of 5-Fu, 5-Fu + 25 μg/mL BAuNPs, and 25 μg/mL Chi/BAuNPs, with high cell viability rates of 96.52 0.015, 84.180.021, and 81.95 0.036%, respectively. After which, the toxic effect was significantly increased with an increment in the 5-Fu + BAuNPs and 5-Fu + Chi/BAuNPs composite concentrations on non-malignant HFs cells, reaching its maximum point by recording 40.14 0.25% cell viability at 5-Fu + 500 μg/mL Chi/BAuNPs, followed by 43.13 0.015 and 51.87 0.025% cell viability rates at 5-Fu + 500 μg/mL BAuNPs and 5-Fu + 200 μg/mL Chi/BAuNPs, respectively, as compared with the 100% cell viability rate in the negative control. The amounts of 5-Fu + Chi/BAuNPs and 5-Fu + BAuNPs composites required to decrease the cell viability rate of HFs non-malignant cells to 50% of the initial population (IC_50_) were 424.52 μg/mL and 416.8 μg/mL, respectively (Figure 9B).

**Figure 10:**
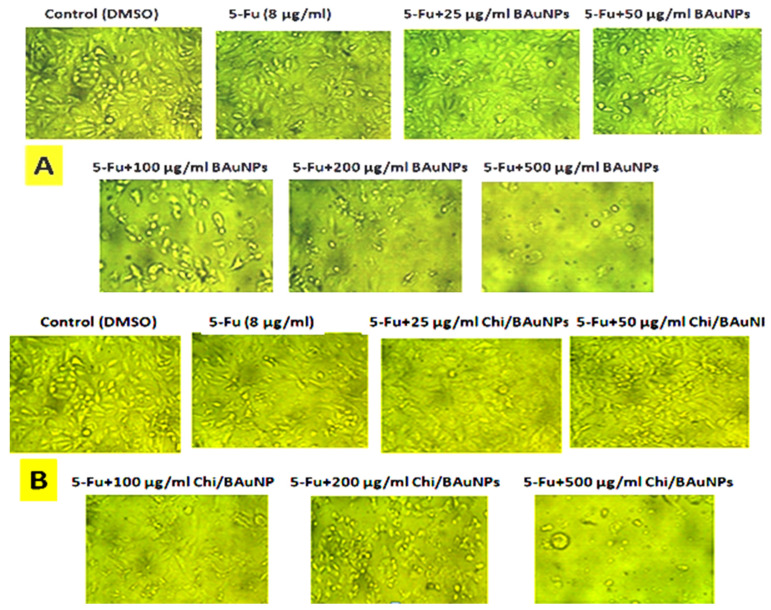
Dose-dependent cell viability effects of different concentrations (0, 25, 50, 100, 200, and 500 µg/mL) of 5-Fu + BAuNPs (**A**) and 5-Fu + Chi/BAuNPs (**B**) composites on human breast adenocarcinoma (MCF7) cell line via MTT assay at 48 h exposure time as compared with negative control and 8 µg/mL free 5-Fu (positive control).

**Figure 11:**
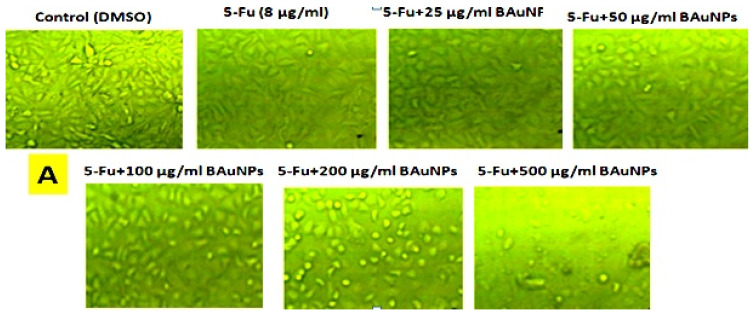

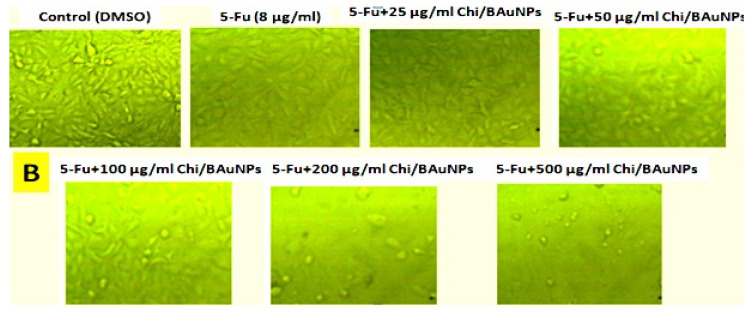
Dose-dependent cell viability effects of different concentrations (0, 25, 50, 100, 200, and 500 µg/mL) of 5-Fu + BAuNPs (**A**) and 5-Fu + Chi/BAuNPs (**B**) composites on non-malignant human fibroblast (HFs) cell line at 48h exposure time via MTT assay as compared with negative control and 8 µg/mL free 5-Fu (positive control).

The obtained results for the cell viability effects of various concentrations (0, 25, 50, 100, 200, and 500 µg/mL) of 5-Fu + BAuNPs and 5-Fu + Chi/BAuNPs composites on the mortality rate and morphological changes of MCF7 cancer cells (Figure 10) and non-malignant HFs cells (Figure 11) that are dose dependent, indicate a significant increase in the mortality rate associated with the large-scale morphological changes that occur at the cell surface. In the cytoskeleton, necrotic cells and apoptotic cells that can be followed and observed were related to the maximum cell cytotoxicity activity of different concentrations of 5-Fu + BAuNPs and 5-Fu + Chi/BauNPs composites against MCF7 cancer cells, i.e., 80.1, 73.07, and 70.58% cell mortality rate at 5-Fu + 500 μg/mL and 5-Fu + 200 μg/mL Chi/BAuNPs (Figure 10A) and 5-Fu + 500 μg/mL BAuNPs (Figure 10B), respectively; while for the maximum cell cytotoxicity activity of different concentrations of 5-Fu + BAuNPs and 5-Fu + Chi/BAuNPs composites against normal HFs cells, we observed a 59.86, 56.87, and 48.73% cell mortality rate at 5-Fu + 500 μg/mL Chi/BAuNPs, 5-Fu + 500 μg/mL 5-Fu + BAuNPs (Figure 11A), and 5-Fu + 200 μg/mL BAuNPs (Figure 11B), respectively.

## 3. Discussion

According to the chemical compositions of the *S. officinalis* aqueous extract, as determined by the GC/MS analysis data presented in Table 1, the *S. officinalis* L. extract was rich in the main bioactive components that demonstrated antioxidant, antimicrobial, antitumor, anti-inflammatory, insecticidal, and hepatic protection of monoterpenes such as thujone and carvacrol, in agreement with previous studies reports [65,66]. A natural terpenoid called thujone fraction, found in many medicinal plants, has antioxidant, anti-diabetic, and anti-tumorigenic characteristics, and it is utilized as a food additive and cosmetic additive [67]. Well-known chemicals with specific antibacterial properties include camphor and 1,8-cineole [68,69]. The flavonoids rosmarinic acid, luteolin, quercetin, and apigenin were identified in the ethyl acetate fraction of *R. officinalis* flowers. The primary components of sage that have been associated with its antibacterial and antioxidant effects include thujone (54.2% *v*/*w*) in the essential oil of *S. fruticosa* species, camphor (6.9%, *v*/*w*), 1, 8-cineole (13.0% *v*/*w*) in the essential oil of *S. ringens* species [70], 1,8-cineole in different samples of *S. officinalis* (ranging between 39.5 and 50.3%), and camphor (10.3–25.0%) [71]. This difference in the sequence of the essential elements may be due to variances in the plant’s natural habitat (land, weather, etc.).

Our results indicated the presence of phenolic compounds in *S. officinalis* extract among the phenolic compounds determined in sage extracts, such as salvianolic acid (20.69%), rosmarinic acid (11.42%), caffeic acid hexoside (10.19%), luteolin-7-o-rutinose (9.78%), apigenin (8.66%), apigenin-7-glucoside (8.12%), dicaffeoylquinic acid (7.76%), carvacrol (6.03%), methyl rosmarenate (4.14%), thujone (2.37%), salvigenin (2.0%), ferulic acid derivative (1.88%), and camphor (0.53%), as well as traces of other phenolic compounds. These results are in agreement with previous studies [72]. The main avonoid and phenolic substances were apigenin and thymol, respectively. More than 25 and 21% of the phenolic chemicals isolated from sage and thyme, respectively, were apigenin and thymol. Such results are in agreement with those reported by Wojdylo et al. [73] and Shan et al. [74]. The improved antioxidant capability of the thyme–sage mixture can be attributed mostly to these components [75]. According to Roby et al. [76], the sage methanolic solution extract’s enhanced DPPH radical scavenging action was probably regulated by flavonoid components such as apigenin.

As a new nanomaterial, the biosynthesis of gold nanoparticles in *S. officinalis* extract as a reducing agent has significant advantages—namely, stability and feasibility—compared with other nanoparticles. Based on their bioavailability, stability, sensitivity, and specificity, the BAuNP nanocomposite was successfully created in the current investigation. TEM is the most efficient method of determining the size and morphological structure of a nanostructure. The TEM image showed that the produced BAuNPs demonstrated good shaping, confirming the spherical Au nanostructures’ crystalline structure and ranging in size from 15 to 70 nm. The measured mean value of the formed BAuNPs composite size was 21.6 nm. In agreement with previous research described by [77,78,79], the gold nanostructures (Au) were associated with multiple distinct peaks in the EDX spectra of (Au), with other subsidiary peaks of the carbon peak attributed to the TEM grid and oxygen peaks attributed to phytochemical traces [50,51,80].

FT-IR analysis was conducted to detect the functional groups responsible for reduction, capping, and stabilizing the synthesized gold nanoparticles. The FTIR spectra of AuNPs revealed absorption peaks at different wave numbers that correspond to many functional groups, such as the C=O, C=N, N-H, C-O, C-N, and C-H stretching vibrations of the alkane group and aldehydic variable group. The obtained functional groups of the FTIR spectra were in agreement with previous studies [79]. The particle size measurement of the particle surface charge was used to assess the conjugate stability of green-synthesized gold nanoparticles in *S. officinalis* extract as reducing agents that have a high content of bioactive fractions of terpenoids, polyphenols, and flavonoids. This is in agreement with previous studies that indicated that nanoparticles are affected by zeta values in solutions, which stabilize nanoparticles if the zeta values are negative [15,79,81,82].

In this work, the inhibitory effect of the prepared BAuNPs and Chi/BAuNPs composites against DPPH and ABTS radicals was assayed based on the composites’ reduction in the presence of an antioxidant compound as a hydrogen donor, and the results are in agreement with previous study [79]. For the determination of MIC, a series of dilutions of green-synthesized BAuNPs and Chi/BAuNPs composites (100, 200, and 300 μg/mL) were evaluated against human pathogenic and multidrug-resistant bacterial strains of *E. coli*, *P. aeruginosa*, *K. pneumonia*, and *S. aureus.*

The results revealed that among the Chi/BAuNPs conjugates, Chi/BAuNPs composites exhibited higher antimicrobial activity against *Pseudomonas aeruginosa*, *E. coli*, *K. pneumonia*, and *S. aureus* compared with BAuNPs. In agreement with [82], gold nanoparticles in different dimensions and shapes are the most widely studied nanomaterials for antibacterial applications [60]. The use of sage extracts in the biosynthesis of gold nanoparticles enhanced the antibacterial activity of the BAuNPs and chitosan-encapsulated AuNPs that related to carvacrol, rosmarinic acid, and apigenin fractions of sage extracts [83].

The cytotoxic effect of a free 5-Fu chemical drug (8 g/mL), and in combination with diverse concentrations (25, 50, 100, 200, and 500 g/mL) of BAuNPs and Chi/BAuNPs composites, was studied in MCF7 cancer cells and non-malignant HFs cells, and the cytotoxicity consequences were analyzed using the MTT assay. The results indicated that the cytotoxicity effect was dose-dependent, while the cell mortality rate increased as the concentrations of nanoparticles increased, relating to the decreased cell viability of the investigated cancer and normal cells. The MCF-7 cancer cells were more sensitive to the cytotoxicity effect of 5-Fu, 5-Fu + BAuNPs, and 5-Fu + Chi/BAuNPs nanocomposites than non-malignant HFs cells, which were not significantly affected by the cytotoxicity effect of the investigated 5-Fu + NPs. Therefore, our study examined the synergistic effect of BAuNPs, chitosan-capped AuNPs, and 5-FU on breast carcinoma MCF7 and non-malignant HFs cell viability. The IC_50_ values were calculated for the cytotoxic effect of 5-Fu + BAuNPs and 5-Fu + chitosan-capped BAuNPs in diverse concentrations against MCF7 cancer cells and normal HFs cells. The results showed that an increase in IC_50_ values in the case of non-malignant cells treated with 5-Fu combined with nanogold and chitosan-capped nanogold is responsible for the non-significant cytotoxic effect of nanocomposites against HFs. Normal cells and the cytotoxic selectivity of nanogold and chitosan-encapsulated AuNPs against cancer cell lines, in addition to the synergistic effect of a combination of gold NPs and chitosan-capped AuNPs with a chemical drug (5-Fu), significantly reduced MCF7 and HFs cell viability at a concentration at which the active drug did not induce an effect. Our results were in agreement with other studies and demonstrate that administering Fe_3_O_4_ NPs in combination with 5-Fu NPs will lower the dosage of the drug needed to produce noticeable antitumor action [84]. The latter outcome is relevant since 5-Fu has a high level of toxicity, which can be decreased, for example, by employing metal–organic frameworks for its immobilization for anticancer activity enhancement [85,86,87].

## 4. Materials and Methods

### 4.1. Chemicals and Reagents

All of the chemicals and reagents used in this investigation met the standards of an analytical laboratory and had purity levels over 98%. DMSO (Sigma-Aldrich, St. Louis, MO, USA), chitosan (MW 100–300 kDa), gallic acid, Folin–Ciocalteu reagents, penicillin–streptomycin antibiotic solution (10,000 U/mL of penicillin and 10,000 U/mL of streptomycin), 5-fluorouracil (5-Fu), and microbial materials such as nutrient agar were bought from Sigma-Aldrich, St. Louis, MO, USA. Loba Chemie (Mumbai, India) was used to acquire chloroauric acid (HAuCl_4_·3H_2_O). The following items were bought from Gibco, Waltham, MA, USA: cell culture media, trypsin, penicillin/streptomycin, andphosphate buffered saline, *N*-Methyl-*N*-(trimethylsilyl)trifluoroacetamide (MSTFA), 99.8% (Sigma Aldrich, USA). Every other substance, including reagents, was of analytical grade.

### 4.2. Plant Materials

Sage (*S. officinalis* L.) plants were collected manually in September 2022 from their natural habitat, the Borg El-Arab city (30°4948.3′ N, 29°3143.5′ E) on the northern Mediterranean coast of Egypt. The collected plants were identified according to Gonz’alez-Tejero et al.’s [88] procedures and confirmed by the botanists at the Botany Department of the Faculty of Science, Mansoura University, Egypt. The aerial parts were washed three times with distilled (dist.) water to remove any undesired matter and dried in an oven at 40–45 °C for five days. They were then pulverized to a fine powder using a lab grinder and sieved using a 63-mm sieve. They were then kept frozen in amber bottles at −18 °C for further tests.

### 4.3. Aqueous Extraction of S. officinalis

Ten grams of the dried leaf sample were combined with 100 mL of double-distilled water (DDW) and heated to 100 °C for 10 min under reduced pressure in order to create an aqueous extract of the *S. officinalis* plant leaf (stock 10%). The produced solution was then sonicated for 15 min and refluxed in a water bath at 90 °C for 2 h. After that, it was ultra-filtered again using a 0.22 µm polyethersulfone membrane filter (TPP, Techno Plastic, Trasadingen, Switzerland).

Samples were derivatized based on the protocol devised by Gullberg and co-workers [89]. Briefly, to each dried sample, 30 μL of 20 mg/mL methoxylamine hydrochloride (98%, Sigma-Aldrich) in pyridine (Rathburn chemicals, Walkerburn, UK) was added. Each sample was vortexed briefly and left to stand at room temperature for 17 h. After the addition of 30 μL of *N*-Methyl-*N*-(trimethylsilyl)trifluoroacetamide (MSTFA) to each sample, they were left to stand at room temperature for one hour. The samples were diluted 1:20 in hexane. A volume of 1 μL of sample was injected, and samples were run splitless.

#### 4.3.1. Chemical Characterization of *S. officinalis* Extract

The major chemical composition of *S. officinalis* leaf aqueous extract was evaluated via a gas chromatography–mass spectrometry (GC-MS) instrument. GC-MS analyses were performed with a GC-MS-QP2010 Ultra analysis system (Shimadzu, Tokyo, Japan). Compounds were separated on a Premier C18 5-micron (2.1 × 100mm reversed-phase C18 column with 120A pore size) using helium as the carrier gas with a constant flow rate of 1.5 mL/min. The oven temperature program was initiated at 50 °C, held for 3 min, then increased at a rate of 8 °C/min to 250 °C and held for 10 min. The spectrophotometer was operated in electron-impact mode. The injector, interface, and ion source were kept at 250, 250, and 220 °C, respectively. Split injection (1 µL diluted sample in n-hexane (1:1, *v*/*v*)) was conducted with a split ratio of 1:20.

Identification of the components of the sample was based on a comparison of their relative indices and mass spectra (RI-MS) via computer matching with WILEY and National Institute of Standards and Technology (NIST08) library data (http://webbook.nist.gov, accessed on 20 November 2021) provided with the computer-controlled GC-MS system. Individually isolated compound identifications were also performed by comparing their mass spectra and retention times with authentic compounds.

#### 4.3.2. Folin–Ciocalteu Assay of *S. officinalis* Extract

For the total concentration of the phenolic compounds in the aqueous extracts of *S. officinalis*, according to the Folin–Ciocalteu (F-C) assay described by Slinkard et al. [90], 300 μL plant extract was taken in a test tube; 1 mL methanol, 3.16 mL distilled water, and 200 μL Folin–Ciocalteu reagent were added; then, after 8 min of incubation at room temperature, we added 2.0 mL of a 7.5% (*m*/*v*) sodium carbonate (Na_2_CO_3_) solution (10%), and the test tube was covered with aluminum foil and incubated at 50 °C for 20 min. A blank was prepared using the same procedure but replacing the plant extract with an equal volume of methanol. The absorbance of the sample was determined using a UV-visible spectrophotometer at 765 nm. The calibration solutions of the gallic acid (GA) standard contained from 10 to 200 mg/L of GA. The final results are expressed as GA equivalents.

### 4.4. Green Synthesis of AuNPs with Aqueous Leaf Extract

For the biosynthesis of gold nanoparticles (BAuNPs), a 3 mM aqueous Au solution was prepared from solid tetrachloroauric acid (HAuCl_4_·3H_2_O). Then, 10.0 mL of 1% (*v*/*v*) *S. officinalis* aqueous extract was directly mixed with 10 mL of gold ion solution, previously prepared by diluting HauCl_4_·3H_2_O in a conical flask wrapped with aluminum foil to prevent reduction with light. Then, we incubated the mixtures in a water bath at 50 °C for 24 h. The mixture’s color changed from colorless to purple, signifying the creation of AuNPs. The suspension that followed was filtered and dried in a vacuum after being cleaned three times with distilled water.

### 4.5. Characterization of BAuNPs and Chi/BAuNPs

#### 4.5.1. UV-Vis Absorption Spectrophotometer

Samples containing the created BAuNPs were subjected to measurements via UV-Vis absorption spectrophotometry at 530 nm using a UV-Vis spectrophotometer (Genway, Yokohama, Japan). The change in mixture color was an indication of gold ion reduction and the creation of green-synthesized AuNPs.

#### 4.5.2. FT-IR

The chemical structure of the created BAuNPs and Chi/BAuNPs was determined using a FT-IR Tensor 27 spectrometer (Bruker, Yokohama, Japan) in the range 4000–400 cm^−1^, with a resolution of 4 cm^−1^.

#### 4.5.3. TEM and Energy-Dispersive X-ray Spectroscopy

A TEM tool was used to assess the surface morphology, size, and shape of the produced BAuNPs conjugate. On carbon-coated copper grids, a drop of a sonicated methanolic solution of BAuNPs or Chi/BAuNPs was applied, and the thin films were subsequently air-dried and examined under a JEM-2100F transmission electron microscope (JEOL, Tokyo, Japan). The lithium-doped silicon EDAX detector, chilled by liquid nitrogen, was used to gather energy-dispersive X-ray spectra.

#### 4.5.4. Particle Size Analysis

The BAuNPs conjugate powder was recorded via Zeta potential analyzer: The Malvern Zetasizer Nano ZS system measures a particle size distribution from 0.6 nm to 6 μm. The used gold solution was freshly prepared.

### 4.6. Synthesis of Chitosan–BAuNPs Conjugates

Chi/BAuNPs were synthesized according to the technique reported by Dananjaya et al. [91] protocol with minimum changes, utilizing chitosan as a reducing and stabilizing agent by chemical reduction process. Chitosan solution (0.2% *w*/*v*) was prepared by dissolving the chitosan having a molecular weight of 375 kDa (Showa, Japan) in 0.01 M acetic acid (Sigma Aldrich, USA) at 65 °C, over 2 hrs with stirring to create a homogeneous solution. Green-synthesized BAuNPs (100 g mL^−1^) were added in a concentration of 10% (*w*/*v*), drop by drop, into a 20 mL chitosan solution previously prepared and stirred at 300 rpm for 5 h at 50 °C to develop the Chi/BAuNPs composite. The BAuNPs and Chi/BAuNPs composites were dissolving in 2% dimethyl sulfoxide (DMSO) at different concentrations (100, 200, and 300 g/mL) for the next bioassay.

### 4.7. Antioxidant Activity of BAuNPs and Chi/BAuNPs

The DPPH scavenging activity of BAuNPs and Chi/BAuNPs composites was estimated according to Zengin et al. [26]. Briefly, a DPPH working solution (1 mg DPPH/10 mL methanol) and serial dilutions (100, 200, and 300 µg/mL) of BAuNPs and Chi/BAuNPs composites were prepared. A total of 1 mL of nanocomposites was diluted with 2 mL of ethanol; then, a DPPH solution was added with a final concentration of 100 μmol/L. The resulting solution was incubated in the dark at 25 °C for 30 min. The assay depends on the reduction of DPPH (purple color) to a diphenyl picrylhydrazine (yellow color), and the remaining DPPH was measured at 517 nm. A control reading was obtained using methanol instead of the extract. The DPPH radical scavenging activity was expressed as a percentage of inhibition activity, and ascorbic acid was used as a positive control.

The percentage inhibition of DPPH∙ was calculated by applying this formula:

DPPH scavenged (%) = [(Ab − As) / Ab] × 100.

Ab: absorbance of blank; As: absorbance of sample

The ABTS scavenging activity of BAuNPs and Chi/BAuNPs composites was estimated according to Zengin et al.’s [92] and Lu and Foo’s [93] protocols based on the ability of antioxidants to reduce radical cation to ABTS− 2. ABTS radical cation solution was produced by reacting 7.0 mM ABTS with K_2_S_2_O_8_ (2.45 mM) at a ratio of 2/1 (*v*/*v*). The mixture could stand in the dark at room temperature for 12 h. After adjusting the pH by treating the ABTS solution with phosphate buffer (0.1 mM, pH 7.4), BAuNPs and Chi/BAuNPs composites were treated with ABTS (1.0 mL) at serial concentrations (100, 200, and 300 µg/mL). The sample absorbance was measured at 734 nm using a UV-VIS spectrophotometer (Genway, Japan), and the ABTS concentration was calculated via the calibration curve. Ascorbic acid was used as a positive control.

The percentage inhibition of DPPH∙ was calculated by applying this formula:

ABTS scavenged (%) = [(Ab − As) / Ab] × 100.

Ab: absorbance of blank; As: absorbance of sample

### 4.8. Antibacterial Assayof BAuNPs and Chi/BAuNPs

#### 4.8.1. Microbial Strains

Three Gram-negative bacteria (*P. aeruginosa* MTCC1034, *E. coli* ATCC 25922, and *K. pneumonia* ATCC 13883) and one Gram-positive bacterium (*S. aureus* ATCC 2592_ were grown in Luria broth medium and incubated at 37 °C for 16 to 18 h [94]. The antibacterial potential of green-synthesized BAuNPs and Chi/BAuNPs was determined via an agar-well diffusion assay [95].

#### 4.8.2. Antibacterial Assay

The antibacterial activity of BAuNPs and Chi/BAuNPs conjugates against human pathogenic and multidrug-resistant bacterial strains of *P. aeruginosa*, *S. aureus*, *K. pneumonia*, and *E. coli* in vitro was carried out via the disc diffusion method following US CLSI (Clinical and Laboratory Standards Institute) [96]. The BAuNPs and Chi/BAuNPs composites were dissolved in 2% DMSO to final concentrations (100, 200, and 300 µg/mL). The solutions were sterilized via filtration on 0.45 µm millipore filters. Then, 20 µL of different concentrations of prepared nanosolutions were soaked into sterile filter paper discs. These discs were placed on Muller–Hinton agar plates, previously swabbed with 100 µL of bacterial inoculum (approximately 10^8^ CFU/mL). The DMSO was used as a negative control, as compared with antibiotic standards of penicillin–streptomycin solution (10 µg/mL) as a positive control for comparison, using a filter paper disc (5 mm) in the assay. The minimal inhibitory concentration (MIC) and minimum bacterial concentration (MBC) tests of BAuNPs and Chi/BAuNPs composites in DMSO were performed via a serial microdilution method according to [97,98].

### 4.9. Anticancer Activity of BAuNPs and Chi/BAuNPs Conjugates

#### 4.9.1. Cell Culture 

The selected cell lines are human breast adenocarcinoma (MCF7) and normal cell lines. Human fibroblasts (HFs) were obtained from the American Type Culture Collection, maintained frozen in liquid nitrogen (−180 °C) at the Tumor Biology Department, National Cancer Institute, Cairo, Egypt. The cell lines were inoculated in a 96-well tissue culture plate at 1 × 10^5^ cells ml^−1^ (100 uL per well) and incubated at 37 °C for 24 h to develop a complete monolayer sheet. Growth medium was decanted from 96-well microtiter plates after a confluent sheet of cells was formed. The cell monolayer was washed twice with wash media.

#### 4.9.2. Evaluation of Cytotoxicity by MTT Assay

An MTT standard cytotoxicity assay was utilized to evaluate the cytotoxic activity of free 5-Fu (8 µg/mL) and different concentrations (90, 25, 50, 100, 200, and 500 µg/mL) of 5-Fu + BAuNPs and 5-Fu + Chi/BAuNPs, as described by Mosmann [99] and Hamida et al. [100], at 48 h exposure time. Two-fold dilutions of the tested sample were made in RPMI medium with 2% serum (maintenance medium), and 0.1 mL of each dilution was tested in different wells, leaving 3 wells as controls and receiving only maintenance medium. The plate was incubated in 5% CO_2_ at 37 °C and examined. Cells were checked for any physical signs of toxicity, e.g., partial or complete loss of the monolayer, rounding, shrinkage, or cell granulation. For each cell line, the cell viability potential and mortality percentages against each concentration and the half-maximal inhibitory concentration (IC_50_) were determined.

### 4.10. Statistical Analysis

The data were presented as the mean ± standard deviations (SD) via triplicate experiments. Statistical analysis was performed with SPSS software 16.0 version (SPSS Inc., Chicago, IL, USA). The obtained data of phytochemical and biological assays findings were analyzed via one-way ANOVA test performed for a significant difference at the *p* < 0.05 level [101]. 

## 5. Conclusions

*S. officinalis* extract, studied for its high phenol and flavonoid content, has demonstrated antioxidant activity and antibacterial and antifungal properties in naturopathic medicine. This research used nanomedicine to biosynthesize gold nanoparticles from *S. officinalis* extract and coat them with safe and environmentally friendly chitosan-coated AuNP conjugates to develop new therapies in response to the increasing interest of today’s society and the pharmaceutical industry in medicinal plants. In recent decades, chitosan has received great interest and attention due to its wide range of potential applications and unique advantages. In this study, nanocomposites based on AuNPs and chitosan were prepared using a simple method. This nanocomposite has promising antibacterial activity against Gram-negative and Gram-positive bacteria. As-prepared BAuNPs and chitosan-coated BAuNPs showed potential antioxidant and antibacterial activities against multidrug-resistant (MDR) and multisensitive (MS) bacterial isolates of human pathogens: *P. aeruginosa*, *E. coli*, *K. pneumonia*, and *S. aureus*. In addition, AuNPs and Chi/AuNPs nanocomposites are safe to use as they show very high toxicity to MCF7 cancer cells and very low toxicity to normal HF cell lines. Our aim is to further investigate the use of gold nanoparticles in medical, pharmaceutical, food, and cosmetic applications to determine their dosage.

## Figures and Tables

**Table 1:** The relative percentage of *S. officinalis* aqueous extracts constituents.

ID	Name of the Compound	Retention Time (min)	Peak Area (%)	Identification *
1	Rosmarinic acid	4.089	11.4228361	RI, MS
2	Sabinene	4.628	1.07748449	RI, MS
3	β-pinene	5.443	0.55135934	RI, MS
4	5-*O*-Caffeoylquinic acids	5.604	0.81992173	RI, MS
5	Quercetin 3-*O*-rhamnoside	5.960	1.29482077	RI, MS
6	Caffeic acid hexoside	17.287	10.1940505	RI, MS
7	Ferulic acid	17.432	1.87677256	RI, MS
8	Dicaffeoylquinic acid	18.532	7.76416171	RI, MS
9	Ferulic acid derivative	19.048	0.58989134	RI, MS
10	Carnosic acid	20.214	0.36857071	RI, MS
11	Salvianolic acid C	20.220	20.6931596	RI, MS
12	Carvacrol	36.884	6.03691661	RI, MS
13	Camphor	38.509	0.53245347	RI, MS
14	Thujone	38.816	2.37399975	RI, MS
15	Methyl rosmarenate	40.611	4.13873184	RI, MS
16	Methyl carnosate	40.89	1.68127316	RI, MS
17	Apigenin	49.700	8.6623548	RI, MS
18	Luteolin-7-o-rutinose	49.916	9.785653	RI, MS
19	Apigenin-7-glucoside	52.649	8.12475124	RI, MS
20	Salvigenin	55.912	2.0040468	RI, MS
21	Total	--	99.993	-

* (RI, MS): Identification of compounds via relative indices and mass spectra.

**Table 2:** The percentage inhibition of DPPH and ABTS free radicals in the presence of different concentrations of green-synthesized AuNPs and Chi/BAuNPs conjugates, using ascorbic acid as a positive control.

Concentrations (μg/mL)		% DPPH	% ABTS
BAuNPs	100	22.60 ± 1.06	17.65 ± 0.15
	200	34.15 ± 4.62	28.21 ± 2.025
	300	51.08 ± 3.86	44.22 ± 4.02
Chi/BAuNPs	100	29.32 ± 2.61	22.46 ± 1.62
	200	54.28 ± 3.05	36.7 ± 1.09
	300	69.84 ± 6.15	53.15 ± 2.45
Ascorbic acid	100	56.22 ± 4.6	36.62 ± 3.06
	200	63.59 ± 6.8	51.82 ± 2.56
	300	81.05 ± 8.41	67.26 ± 3.87

**Table 3:** Antimicrobial activity of BAuNPs and Chi/BAuNPs composites against four bacterial strains with the indicated concentrations. Values are expressed as mean ± SE (n = 5).

Concentrations (µg/mL)		Inhibition Zone (mm)			
		*P. aeruginosa*	*E. coli*	*K. pneumonia*	*S. aureus*
BAuNPs	100	17 ± 0.012	11 ± 0.012	12 ± 0.014	14 ± 0.014
	200	21 ± 0.15	14 ± 0.15	14 ± 0.12	16 ± 0.012
	300	28 ± 0.15	19 ± 0.16	20 ± 0.15	21 ± 0.13
Chi/ BAuNPs	100	19 ± 0.13	18 ± 0.14	19 ± 0.021	21 ± 0.15
	200	24 ± 0.12	23 ± 0.15	26 ± 0.12	27 ± 0.012
	300	30 ± 0.25	28 ± 0.11	29 ± 0.14	31 ± 0.024
Penicillin/streptomycin	10	33 ± 0.13	30 ± 0.16	31 ± 0.18	32 ± 0.02

**Table 4:** MIC and MBC values of BAuNPs and Chi/BAuNPs composites against four human pathogenic bacterial strains with the indicated concentrations. Values are expressed by mean ± SD (n = 5).

Concentrations (µg/mL)		Microorganisms			
		*P. aeruginosa*	*E. coli*	*K. pneumonia*	*S. aureus*
BAuNPs	MIC	984	648	843	1352
	MBC	546	386	406	617
Chi/ BAuNPs	MIC	563	394	453	711
	MBC	324	186	213	412
Penicillin/streptomycin	MIC	36	12	16	43
	MBC	16	8	10	19

## Data Availability

Data are contained within the article.
